# Advances, challenges and future applications of liver organoids in experimental regenerative medicine

**DOI:** 10.3389/fmed.2024.1521851

**Published:** 2025-01-24

**Authors:** Da Gong, Jiaye Mo, Mei Zhai, Fulin Zhou, Guocai Wang, Shaohua Ma, Xiaoyong Dai, Xuesong Deng

**Affiliations:** ^1^Department of Hepatobiliary Surgery, the First Affiliated Hospital of Shenzhen University, Health Science Center, Shenzhen Second People’s Hospital, Shenzhen, China; ^2^Guangxi University of Chinese Medicine, Nanning, China; ^3^Department of Clinical Medicine, Guizhou Medical University, Guiyang, China; ^4^Department of Physiology, School of Medicine and State Key Laboratory of Bioactive Molecules and Druggability Assessment, Jinan University, Guangzhou, China; ^5^Institute of Biopharmaceutical and Health Engineering, Shenzhen Key Laboratory of Gene and Antibody Therapy, State Key Laboratory of Chemical Oncogenomics, Tsinghua University Shenzhen International Graduate School, Guangdong, China

**Keywords:** liver organoids, regenerative medicine, liver injury, liver research model, microfluidic and 3D print, organoids-on-a-chip

## Abstract

The liver is a vital organ responsible for numerous metabolic processes in the human body, including the metabolism of drugs and nutrients. After liver damage, the organ can rapidly return to its original size if the causative factor is promptly eliminated. However, when the harmful stimulus persists, the liver’s regenerative capacity becomes compromised. Substantial theoretical feasibility has been demonstrated at the levels of gene expression, molecular interactions, and intercellular dynamics, complemented by numerous successful animal studies. However, a robust model and carrier that closely resemble human physiology are still lacking for translating these theories into practice. The potential for liver regeneration has been a central focus of ongoing research. Over the past decade, the advent of organoid technology has provided improved models and materials for advancing research efforts. Liver organoid technology represents a novel *in vitro* culture system. After several years of refinement, human liver organoids can now accurately replicate the liver’s morphological structure, nutrient and drug metabolism, gene expression, and secretory functions, providing a robust model for liver disease research. Regenerative medicine aims to replicate human organ or tissue functions to repair or replace damaged tissues, restore their structure or function, or stimulate the regeneration of tissues or organs within the body. Liver organoids possess the same structure and function as liver tissue, offering the potential to serve as a viable replacement for the liver, aligning with the goals of regenerative medicine. This review examines the role of liver organoids in regenerative medicine.

## Introduction

The liver organoids are laboratory-engineered models that replicate the liver’s structure and functions using three-dimensional culture and stem cell technologies. Despite their promising potential in medical research and clinical applications, liver organoids face several challenges. First, current liver organoids models often fail to fully replicate the structural and functional complexity of the adult liver, limiting their application in long-term drug testing and disease research. Second, improving the maturity and functionality of liver organoids—particularly their metabolic capacity and immune response—remains a critical challenge. Additionally, challenges such as large-scale production, standardized culture conditions, and the adaptability of liver organoids to individual human variations must be addressed for future clinical applications. In view of the above issues, this review discusses the future development and application prospects of liver organoids in experimental regenerative medicine.

The liver is a vital organ in the human body that is responsible for detoxification, digestion, and nutrient metabolism. Common liver diseases include viral and nonviral hepatitis, hepatic hemangioma, cirrhosis/fibrosis, traumatic liver injury, and liver cancer (1). The adult liver possesses a robust regenerative capacity, allowing it to rapidly restore function following damage (2). Even when a portion of the liver is removed, the remaining liver cells can swiftly proliferate to restore the organ’s volume and function (3). In mammals, when 75 to 85% of the liver is surgically removed, the remaining portion cannot compensate for systemic metabolism, often resulting in fatal posthepatectomy liver failure (PHLF) (4, 5). Chronic damage to the liver typically reduces its regenerative capacity over time (6, 7). Organoid technology has garnered significant attention since its initial discovery (8). Liver organoids, which are three-dimensional cell clusters that mimic the structure and function of the liver, have become a focal point in drug development, metabolism studies, safety assessments, and precision medicine (9–11). Regenerative medicine involves the development of functional tissues and materials to repair or replace damaged organs and tissues or to promote the regeneration of tissues and organs through biological, chemical, and physical means (12). Undoubtedly, the emergence of liver organoids represents a powerful experimental model and a potential tool in regenerative medicine.

## The research model in liver injury

Liver injury can be categorized as either acute or chronic. Acute liver injury often results from traumatic liver ruptures or nontraumatic causes, such as drug-induced liver injury and acute hepatitis A. Chronic liver injury is predominantly caused by hepatitis viruses or autoimmune diseases (13). Traumatic liver injury is relatively common, with numerous cases reported annually (14). Due to the liver’s abundant blood supply and bile secretion, liver injury can lead to shock and acute peritonitis (15). Timely blood volume support and surgical intervention are effective treatments for this type of injury (16). Nontraumatic liver injury often occurs after the consumption of hepatotoxic substances or drugs or following hepatitis A virus infection within a short period (17, 18). Clinically, nontraumatic liver injury is characterized by upper abdominal pain, gastrointestinal symptoms, such as nausea and vomiting, and elevated transaminase and bilirubin levels in biochemical tests (19). Most individuals achieve a favorable prognosis by avoiding hepatotoxic agents and using hepatoprotective drugs, such as glutathione.

The hepatitis B virus (HBV) is the most common cause of chronic liver injury (20). The prevailing view is that long-term HBV infection inhibits virus-specific T-cell function and downregulates innate immune signaling pathways, enabling the virus to evade immune surveillance (21). However, high-level viral replication and the production of subviral hepatitis B surface antigen (HBsAg) particles play crucial roles in promoting liver damage (22). Chronic liver damage leads to edema and fatty degeneration of hepatocytes, triggering persistent low-grade inflammation (23). This is often accompanied by transient high-grade inflammation and the activation of fibrogenic processes, ultimately resulting in viral liver fibrosis (24). Currently, the primary method for preventing hepatitis B virus infection is the administration of the hepatitis B vaccine. Clinical treatment primarily involves the use of interferons and antiviral drugs, although a standardized treatment protocol has not yet been established.

As liver fibrosis progresses, the liver’s basic structure is gradually compromised, eventually leading to irreversible cirrhosis (25). Cirrhosis is a prevalent global disease with common causes including hepatitis B virus infection, excessive fat and lipid intake, and autoimmune hepatitis (26). The main symptoms include liver dysfunctions, such as portal hypertension, ascites, and hypoalbuminemia (27). Treatment focuses on addressing the underlying cause and managing complications, with liver transplantation a viable option in certain cases (27, 28). As cirrhosis advances, regenerative disorders may arise, potentially progressing into hepatocellular carcinoma (29, 30), which is the eighth most prevalent cancer and the third leading cause of cancer-related deaths globally (31).

In the early stages of liver disease, symptoms may be subtle or entirely absent. Space-occupying liver lesions are frequently discovered incidentally during routine physical examinations. Diagnosis typically involves a combination of blood alpha-fetoprotein (AFP) levels, enhanced computed tomography (CT), Magnetic resonance imaging (MRI), and ultrasound angiography (32, 33). Treatment options, depending on the stage of the disease and the patient’s specific condition, include surgical resection, local ablation, interventional therapy, pharmacotherapy, or liver transplantation (34). Unfortunately, the prognosis remains poor even with these treatments (35). Hepatitis B virus infection, cirrhosis, and hepatocellular carcinoma are closely interconnected, forming a critical disease (36). Hepatitis B virus infection is a leading cause of cirrhosis and hepatocellular carcinoma, particularly in Asia (37). Cirrhosis represents a critical intermediate stage in the progression to hepatocellular carcinoma, which is the most severe outcome for patients with cirrhosis (36). For patients with advanced cirrhosis and hepatocellular carcinoma, liver transplantation is a viable treatment option that can significantly enhance survival and quality of life (27, 38, 39).

**Figure 1:**
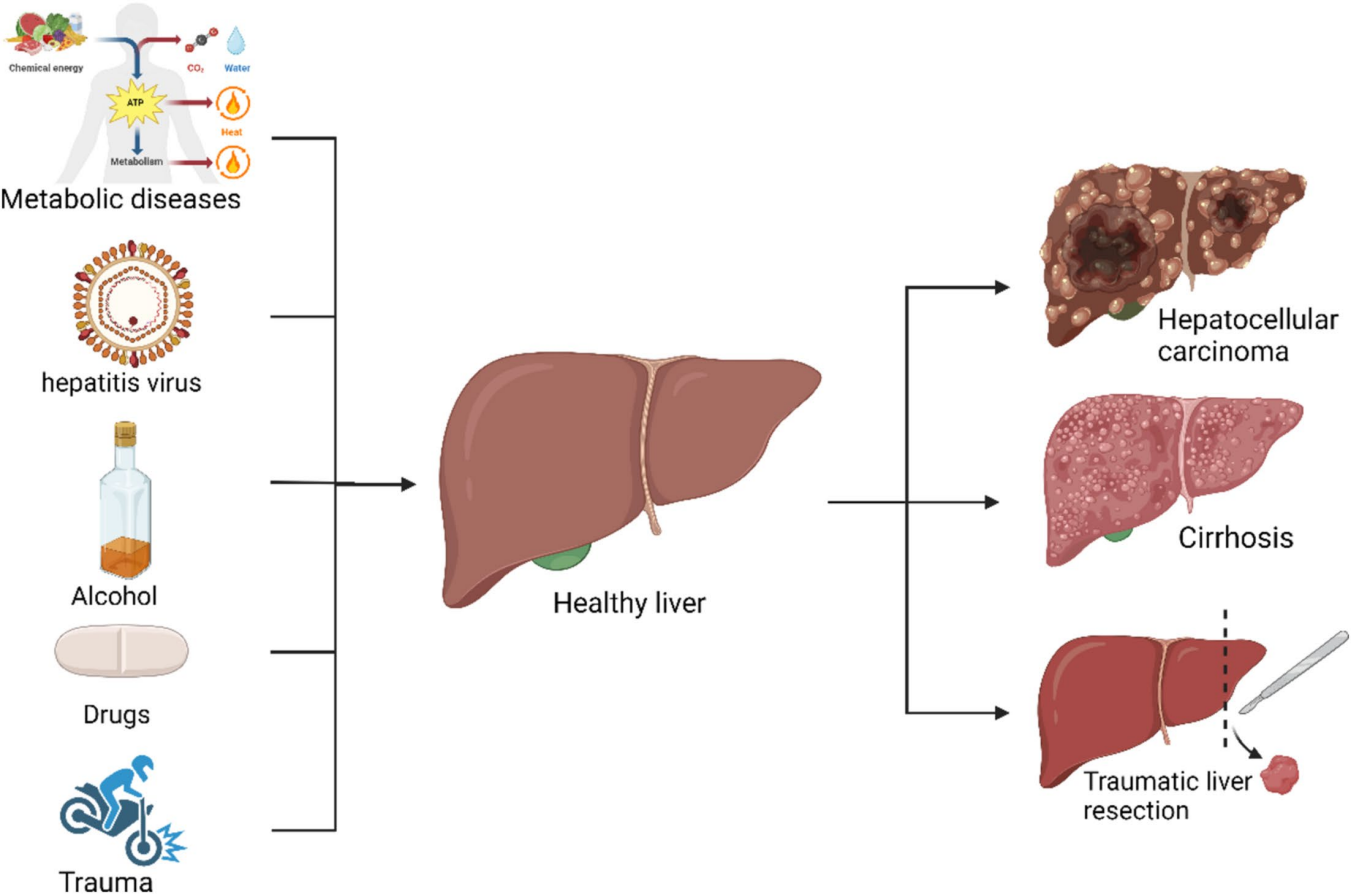
Common factors contributing to liver injury and their consequences: autoimmune diseases, viral hepatitis, alcohol, drugs, and trauma can cause cirrhosis, hepatocellular carcinoma, and acute liver failure.

Liver damage may also result from immune-mediated and genetic disorders. Immune-mediated liver damage, such as autoimmune hepatitis, occurs when the immune system mistakenly targets liver cells, potentially leading to chronic inflammation and significant hepatic injury (40). Primary biliary cholangitis and primary sclerosing cholangitis are additional immune-mediated liver diseases that affect the bile ducts, potentially causing cholestasis and hepatic damage (41). Conversely, hereditary liver diseases, such as hemochromatosis, Wilson’s disease, and α1-antitrypsin deficiency, result from genetic mutations inherited from parents (42, 43). These genetic mutations impair the liver’s ability to metabolize specific substances, leading to their accumulation and subsequent liver damage. For instance, Wilson’s disease involves defective copper metabolism, whereas α1-antitrypsin deficiency impacts protease activity (42, 44). Despite their relative rarity, these disorders significantly affect patient health (45). Additional causes of liver damage include alcoholic liver damage, nonalcoholic fatty liver disease (NAFLD) due to fat accumulation, and drug-induced liver injury (Figure 1). As of 2023, liver diseases have resulted in over 2 million deaths globally, with an increasing trend (46). All of these conditions, with the exception of traumatic liver damage (which involves fewer affected areas), contribute to a reduction in liver regenerative capacity (47).

**Figure 2:**
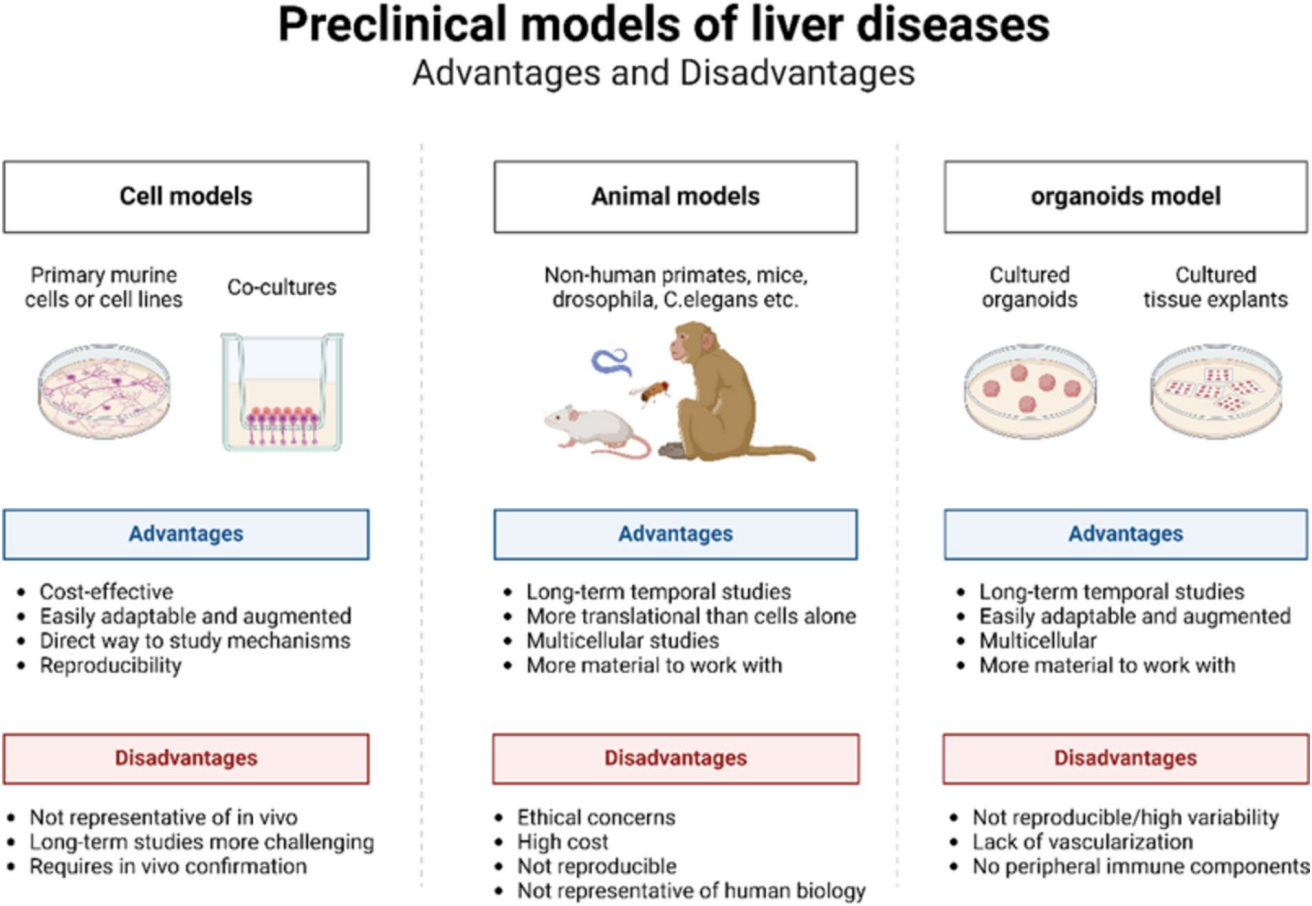
Advantages and disadvantages of cell models, animal models, and organoid models.

Traditionally, animal models and *in vitro* cell models have been extensively utilized to investigate liver diseases (Figure 2) (48). Animal models closely mirror the physiological metabolic processes, tissues, and microenvironment of the human body (49). These models aid in elucidating the pathogenesis of liver diseases, assessing drug efficacy, and investigating novel treatments. Among the various models used for liver diseases, the mouse model is the most prevalent. Models of cirrhosis and fibrosis are typically induced through bile duct ligation, thioacetamide administration, or high-concentration alcohol infusion into the hepatic artery (28, 50). Liver cancer models are commonly established using methods such as chemical induction, dietary modification, genetic engineering, heterotopic transplantation, and humanized mouse systems (51). Numerous construction methods and animal models exist, although deviations are common due to species differences and variations in disease progression (52). The long duration, high cost, and ethical constraints associated with animal models limit the feasibility of short-term, homogeneous, and high-throughput experiments (48).

Traditional 2D cell models, which involve culturing single layers of cells, are straightforward to culture and grow rapidly (53, 54). However, they are confined to a 2D structure consisting of a single layer of cells adhering to a culture medium. These models lack the matrix and immune cells essential for regulating cell behaviors, such as differentiation, survival, proliferation, and migration, making it impossible to accurately simulate the organ structure (55). Consequently, 2D models fail to provide the varied levels of oxygen and nutrients required for multidimensional cell behavior (52). Furthermore, repeated subcultures often lead to the loss of the original cell characteristics (56, 57). As a central organ for food, drug, and energy metabolism, the liver is characterized by a complex and multilayered structure, thus posing significant challenges for 2D cell models. While 2D models can offer valuable insights, they cannot fully replicate the complexity and multidimensional nature of human liver disease.

## The advances in liver organoids

**Figure 3:**
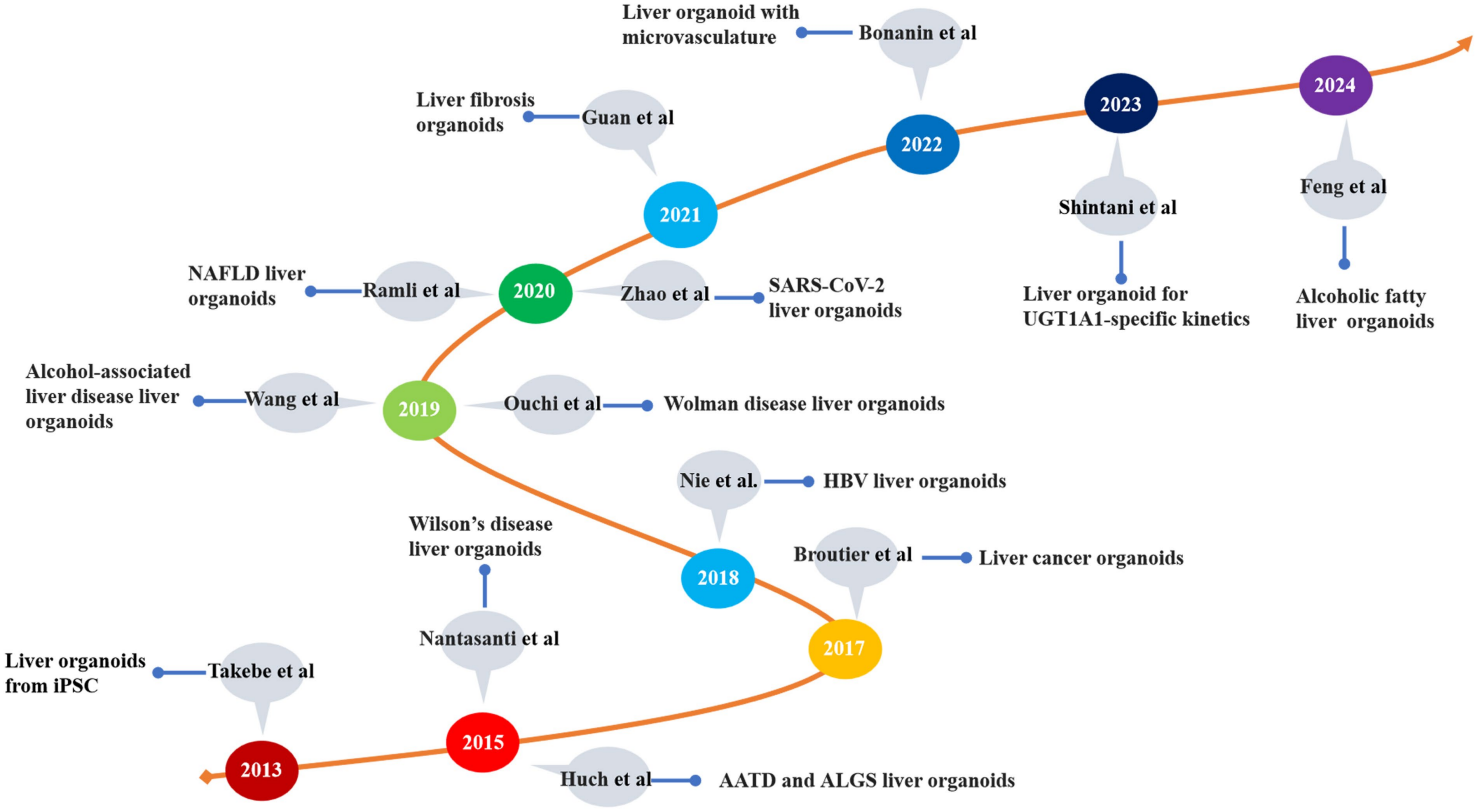
The development of liver disease models. iPSC, induced pluripotent stem cell; AATD, alpha-1 antitrypsin deficiency; ALGS, Alagille syndrome; HBV, hepatitis B virus; NAFLD, nonalcoholic fatty liver disease; UGTIA1, UDP glucuronosyltransferase family 1 member A1.

The organoids represent a novel technology that has emerged over the past decade (58). These models exhibit a three-dimensional tissue structure and retain functions and characteristics similar to those of the original tissue (59). The development history of liver organoids is summarized in Table 1 and Figure 3. Clevers et al. (60) successfully cultured intestinal organoids from Lgr^+^ intestinal stem cells. The authors utilized pluripotent adult stem cells with high plasticity and self-renewal capacity to explore methods for simulating natural growth conditions, precisely controlling growth factors and the cellular microenvironment and guiding stem cells to differentiate along specific pathways to form organoid structures (61). Huch et al. (62) cultured liver organoids from damaged Lgr5^+^ bile duct cells and demonstrated that these organoids maintained the original characteristics of the liver even after long-term proliferation. A subsequent study indicated that liver organoids preserve most of their characteristics even during extended proliferation and passage (63). Skardal et al. (64) developed an *in vitro* model of colorectal cancer liver metastasis. Leite et al. (65) established an organoid model of liver fibrosis. In 2017, they successfully established primary liver tumor (PLC) organoids and showed that these organoids retained the characteristics and heterogeneity of liver cancer (66). Ouchi et al. (67) developed an organoid model of nonalcoholic fatty liver disease (NAFLD) in 2019. In the subsequent year, Elbadawy et al. (68) created a mouse-derived organoid model for nonalcoholic steatohepatitis (NASH). Later, De Crignis et al. (69) successfully cultured liver organoids infected with the hepatitis virus. Organoid models for various liver diseases, such as liver fibrosis, drug-induced liver injury, and glycogen storage disease type Ia (GSD1a), have now been developed (70–72). Based on the number of passages, organoids are classified into short-term and long-term. Short-term organoids are cultured for up to 30 generations or 3 months and are primarily used for low-throughput drug screening, biomarker identification, and the exploration of drug sensitivity and resistance mechanisms (73). In contrast, long-term organoids are suited for high-throughput drug screening and the development of organoid databases (74, 75).

**Table 1:** The development history of liver organoid models.

Authors	Time	Cell source	Result
Clevers et al. (62)	2013/02	Mice derived	The first liver organoids
Skardal et al. (64)	2015/10	Human derived	*In vitro* model of liver metastasis of bowel cancer
Leite et al. (65)	2016/02	Human derived	Detection of drug-induced liver fibrosis in liver organoid models
Huch et al. (66)	2017/12	Human derived	Establishment of models of liver cancer organoids
Vyas et al. (187)	2018/12	Human derived	A composite organoid model of hepatocytes and bile cells
Ouchi et al. (67)	2019/08	Human iPSC	Organoid modeling of NAFLD
Soroka et al. (188)	2019/09	Human derived	Organoid models of biliary cirrhosis
Gómez-Mariano et al. (189)	2020/01	Human derived	The organoid model that α-1 organoid model of antitrypsin deficiency liver disease
Elbadawy et al. (68)	2020/04	Mice derived	Effect of mouse NASH organoid models
De Crignis et al. (69)	2021/07	Human derived	Viral B hepatitis liver organoid model
Guan et al. (70)	2021/10	Human iPSC	Liver organoids for liver fibrosis were successfully established
Li et al. (190)	2022/01	Human derived	Viral C hepatitis liver organoid model
Bonanini et al. (99)	2022/11	Human derived	The microvascular system of liver organoids was constructed
Zhang et al. (72)	2023/05	Human iPSC	Liver organoids have the ability to mimic drug-induced liver injury
Shintani et al. (191)	2023/08	Human iPSC	A liver organoid model for UGT1A1-specific kinetics and toxicity evaluation
Feng et al. (192)	2024/05	Human iPSC	Organoid model of alcoholic fatty liver disease from iPSCs

**Figure 4:**
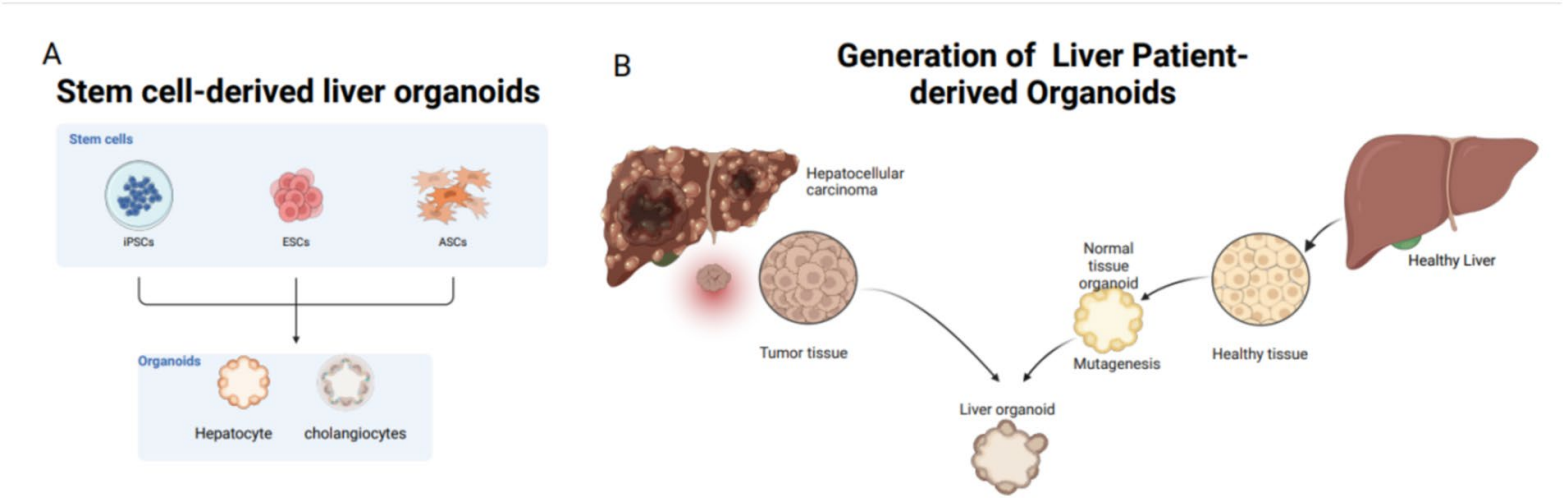
Common tissue sources for liver organoids: stem cells and adult cells. Stem cells include iPSCs, ESCs, and ASCs; adult cells mostly come from patients. **(A)** Sources of stem cells for liver organoids: from ECS, iPSC, ASCs. **(B)** Patient derived, mainly from liver tissue removed after clinical surgery.

Organoids are categorized based on their cell sources into two main types: tissue-derived organoids and pluripotent stem cell-derived organoids, including adult stem cells (ASCs), pluripotent stem cells (PSCs), and patient-derived organoids (PDOs). PSCs are subclassified into embryonic stem cells (ESCs) and induced pluripotent stem cells (iPSCs) (Figure 4) (76). Patient-derived organoids (PDOs) are sourced from mature tissues and preserve the morphological, physiological, and genetic characteristics of the original tissues (77). These organoids are relatively straightforward and require less time to culture and exhibit high cell maturity (78, 79). In 2017, three types of liver tumor organoids—hepatocellular carcinoma, cholangiocarcinoma, and mixed-cell carcinoma—were established from eight liver tumor patients, retaining the morphological and physiological characteristics of the original tumors (66). The following year, Nuciforo et al. (80) utilized tumor needle biopsy technology to generate liver tumor organoids from biopsy samples of hepatocellular carcinoma (HCC) patients, preserving the histological, transcriptomic, and genetic characteristics of the original tumors. While Wu et al. (81) expanded circulating tumor cells (CTCs) from 41 liquid biopsy samples of 31 pancreatic ductal adenocarcinoma (PDAC) patients to develop CTC-derived organoids *in vitro*, there are currently no reports of successfully inducing malignant liver tumor organoids from CTCs. Nevertheless, mature liver tissue can still be used to establish liver organoids, even after extended cryopreservation (82).

PSC-derived organoids originate from ESCs or iPSCs. Cells such as skin fibroblasts are first reprogrammed into pluripotent stem cells in a culture medium and subsequently differentiated into liver cells for organoid culture (83). Since the first iPSCs were generated from adult mouse cells in 2006 (84), subsequent studies have demonstrated the applicability of iPSC technology to human cells (85). CRISPR/Cas9 technology has been employed to overexpress the c-Myc gene, successfully inducing HCC organoids from human liver cell organoids (86). Adult cells from human sources are reprogrammed into iPSCs through the knockout of specific transcription factors, followed by differentiation into liver cells. Although the process is time-consuming, the resulting organoids possess a complex array of cellular components, including mesenchymal, epithelial, and potentially endothelial cells (87). Compared to patient-derived organoids, PSC-derived organoids are particularly useful for studying the early stages of human organ development (88). Additionally, organoids can be derived from human tissues that difficulty to obtain, such as the brain, heart, and bone marrow. Currently, the availability of tissues for establishing liver organoids is increasing, and the associated materials are becoming more accessible.

## The technology in liver organoids

The production of liver organoids has become relatively advanced. The main steps are as follows: tissue is digested into single cells using digestive enzymes (e.g., collagenase, DNAse, and dispase) filtered through a sieve and mixed with matrix gel to form microspheres. Subsequently, signal molecules and biological agents that simulate stem cell growth factors required by stem cells are added. For instance, hepatocyte growth factor (HGF), epidermal growth factor (EGF), fibroblast growth factor (FGF), and Wnt family member 3A (Wnt3A) promote cell proliferation, while dexamethasone, bone morphogenetic protein (BMP), and other cytokines induce hepatocyte maturation. Additionally, mTeSR facilitates the differentiation of bile duct cells (62, 89). In recent years, advancements across multiple disciplines have driven innovation in organoid technology.

Microfluidics is a technology that utilizes microchannels ranging from tens to hundreds of micrometers to process and manipulate small volumes of fluid. This technology can precisely control the cellular environment in fields such as biology and biomedical engineering (90). Microfluidic technology allows for precise control over organoids and provides dynamic physical conditions that facilitate the production of high-throughput, uniform organoid microspheres (91). Organoid microspheres produced using this technology more accurately replicate the *in vivo* liver microenvironment and the interactions between the liver organ model and other tissues and organs (92). This approach holds significant potential for disease simulation, personalized medicine, drug screening, and regenerative medicine (93). Organ-on-a-chip technology employs microfabrication techniques to create a micromodel of a biological organ on a chip-sized device (94). The integration of organoid and organ-on-a-chip technologies results in organoid chips (organoids-on-a-chip). By incorporating microfluidic technology, these chips enable the construction of more precise and controllable organoid models that simulate the microenvironment, vascularization, and tissue interactions (95). Microfluidic liver organoid chips are designed to simulate the *in vivo* liver sinusoid structure and the liver microphysiological environment. This approach partially compensates for the limitations in the structure, composition, and mechanical microenvironment observed in conventional liver organoids (96–98). Co-culturing vascular endothelial cells with liver cells on the chip simulates a three-dimensional vascular network, potentially offering an *in vitro* alternative to animal models and conventional transplantation (99).

3D bioprinting is a branch of 3D printing that uses cells and an extracellular matrix (ECM) as raw materials to construct the desired biological structures (100). This technology can directly print complex tissue or organ structures and has garnered significant attention across various medical fields (101). The primary methods of 3D bioprinting include inkjet, extrusion, laser-assisted bioprinting (LAB), and acoustic-assisted bioprinting (102). This technology has been employed to print various tissues, including skin, heart, bone, cartilage, liver, lung, nerve, and pancreatic tissues (103). 3D bioprinting plays a crucial role in drug screening, disease modeling, and liver regenerative medicine (104, 105). Organoids possess complex biological components and structures, including inherent 3D architecture (59). The synergy between organoids and 3D bioprinting represents a highly promising approach. Currently, the requirements for bioink composition are more stringent than those for 2D cell cultures. Bioink is a specialized material used in bioprinting that supports living cells and other biological components, providing a matrix for cell growth and organization (106, 107). Liver endothelial cells can be layered and printed into a composite organoid model, demonstrating a well-developed vascular and bile duct system, thereby maximizing liver function (108). Using multicellular 3D droplets, HepaRG/HUVECs/LX-2 liver organoids with biomimetic lobule structures have been constructed, effectively simulating cell heterogeneity, spatial organization, and ECM characteristics (109). Current methods for constructing liver organoids still face challenges related to cell heterogeneity, tissue structure, and the integrity of functional components. Therefore, developing liver organoids using integrated design strategies and multidisciplinary engineering methods is paramount.

### The application of liver organoids in liver regeneration

The regenerative capacity of a healthy liver is robust. Even after a major hepatectomy, it can regenerate to its original size within a relatively short period (110). The number of hepatocytes and their sizes both increase, while the number of hepatic lobules decreases (3). In chronic liver injury, the liver’s regenerative capacity becomes unbalanced due to cell damage, repair processes, extracellular matrix deposition, and subsequent structural alterations (111, 112). In viral hepatitis and immune-mediated liver disease, chronic injury is characterized by ongoing repair and regeneration and leads to an imbalance between these two processes over time (113). Ultimately, fibrous tissue repair compresses hepatocyte space, inhibits liver regeneration, and results in cirrhosis characterized by diffuse liver fibrosis, pseudolobules, and regenerative nodules (114). This progression can lead to end-stage liver diseases, including portal hypertension and hepatocellular carcinoma (115, 116).

Regenerative medicine has been a field of exploration for many years. It has evolved from a search for regenerative tissues or organs to methods that stimulate the regeneration of an individual’s own tissues or organs (12). Bone and tooth regeneration techniques are currently well developed (117, 118). However, progress in liver regeneration remains relatively slow. Liver organoids hold a significant position and offer substantial potential in regenerative medicine (Table 2).

**Table 2:** Current status of liver organoid transplantation.

Author	Time	Cell source	Animal	Position	Result
Ye et al. (193)	2016/06	PDO	Mice	Subcutaneous	Hybrid hepatobiliary organoids increase blood albumin concentration
Zhou et al. (194)	2017/05	PDO	Mice	Orthotopic	Orthotopic transplantation of mouse liver organoids
Nie et al. (128)	2018/01	PSCs	Mice	Kidney capsule	Liver organoid transplantation improves the survival rate of mice with acute liver injury
Wu et al. (195)	2019/06	PDO	Mice	Subcapsular spleen	Hybrid hepatobiliary organoids maintain liver function in mice
Tsuchida et al. (131)	2019/12	Mice derived liver	Mice	Portal vein	Portal vein liver organoid transplantation has safety and efficacy
Tsuchida et al. (196)	2020/01	PSCs	Porcine	Portal vein	Human iPSC-derived liver organoids can be safely transplanted
Kruitwagen et al. (197)	2020/02	Dog derived liver	Dog	Portal vein	Extend the lifespan of COMMD1-deficient dog
Sampaziotis et al. (198)	2021/01	PDO	Mice	Intrahepatic ducts	Extends lifespan of mice with bile duct disease
Guo et al. (83)	2021/02	PSCs	Mice	Central nervous system	Understanding the mechanisms of liver failure in DGUOK mutant MDS
Zhang et al. (199)	2021/10	Porcine biliary	Porcine	Patch transplant	Without evidence of emboli or ectopic cell distribution
Salas-Silva et al. (136)	2023/12	PDO	Mice	Submesenteric	Can improve survival rate of mice with acute and chronic liver injury
Li et al. (200)	2024/01	PSCs	Monkey	Liver subcapsular and submesenteric	Effective and safe for advanced liver disease
Yuan et al. (127)	2024/04	PDO	Mice	Intraperitoneal	Encapsulated liver organoids improve survival in mice with acute liver injury

When the liver is injured or a significant portion is removed, the remaining liver may temporarily be unable to compensate, potentially leading to post-hepatectomy liver failure (PHLF), a serious complication and cause of death following liver surgery (119). Clinicians use artificial liver blood purification systems (ALBPS) as extracorporeal temporary replacement therapy, analogous to extracorporeal membrane oxygenation (ECMO) systems. These systems provide time for the remaining liver to regenerate; however, their treatment efficacy remains limited (120). Artificial livers are extracorporeal devices designed for detoxification and metabolic functions (121). They play a pivotal role in treating liver failure and are recommended as a primary option for patients with this condition (122). These devices can be broadly categorized into two types, nonbioartificial livers (NBALs) and bioartificial livers (BALs), which can also be combined to form hybrid artificial livers (HALs) (123). Nonbioartificial livers operate primarily by purifying blood through techniques such as plasma exchange (PE), hemodialysis (HD), hemofiltration (HF), and hemoperfusion (HP)/plasma perfusion (PP).

These devices focus on the detoxification and filtration of metabolic wastes using systems such as the molecular adsorption recirculating system (MARS), fractionated plasma separation and adsorption, and therapeutic plasma exchange (TPE) (124). A bioartificial liver primarily consists of functional hepatocytes within an *in vitro* bioreactor. Hepatocytes perform metabolism, synthesis, and secretion either through blood flow across a semipermeable membrane or by direct contact. These devices are typically used for short-term support (125). Compared to NBALs, BALs are composed of functional hepatocytes that provide essential liver functions, including detoxification, metabolite synthesis, and biotransformation (126). Hybrid artificial livers integrate both approaches: the nonbiological component handles liver detoxification, while the bioartificial component performs detoxification, synthesis, and biotransformation. This combination offers complementary benefits and holds promise for future advancements. Addressing the challenges of stability and long-term functionality in bioartificial livers could potentially be enhanced by integrating liver organoids.

Organoid transplantation has emerged as a prominent topic in regenerative medicine and has shown promising results in animal experiments (Table 2). Yuan et al. (127) transplanted proliferating human hepatocytes (ProliHHs) encapsulated in organoids into the peritoneal cavity of mice with PHLF. This approach aimed to enhance liver regeneration, reduce endotoxins and hyperammonemia, alleviate hypoglycemia, and improve overall survival (127). Functional liver organoid (LO) tissue generated from the endoderm, endothelial cells (EC), and mesenchymal cells (MC) derived from human-induced pluripotent stem cells (hiPSCs) from a single donor can significantly enhance the survival rate and liver function of mice with acute liver injury (128). Such liver organoid grafts need to survive for approximately 1 week to allow the liver to regenerate sufficiently to support systemic metabolic functions. To address this need, Labour et al. (129) developed a structurally adjustable, animal-free, three-dimensional porous scaffold that evenly distributes cells and supports their survival for over 7 days. When combined with other advancements, this scaffold has potential for clinical application.

In chronic liver damage, such as viral hepatitis, advanced disease stages involve concurrent damage, repair, and regeneration. The resulting fibrous tissue compresses functional liver cells, leading to liver failure and symptoms such as ascites and spider nevi (130). In a rat model of chronic liver injury, liver organoid transplantation via the portal vein significantly improved bile reconstruction rates and the replacement of damaged liver tissue. Additionally, ductal reactions were reduced, and precancerous lesions marked by placental glutathione S-transferase (GST-p) were diminished (131). In cases of portal hypertension caused by cirrhosis, transjugular intrahepatic portosystemic shunt (TIPS) and portal vein shunt surgeries can alleviate symptoms such as ascites and hematemesis (132). However, these treatments are ineffective against hepatic encephalopathy caused by hyperammonemia, which can only be managed by restricting protein intake and using medications (133). Combining 3D printing technology with liver organoids to replace liver cell functions can facilitate the degradation of toxic substances, thereby alleviating hepatic encephalopathy (134). Compared to hepatocyte-derived artificial livers, liver organoids offer more precise regulation of biomarkers such as Aspartate aminotransferase (AST), Alanine transaminase (ALT), and blood ammonia (135, 136).

Due to trauma, the liver sustains significant damage. Following emergency surgery, the remaining liver tissue is insufficient to meet the metabolic demands of the body. In such cases, the primary clinical treatment involves providing artificial liver support while waiting for the liver to regenerate to a level that meets the body’s minimum requirements (137). Liver organoids may assist in repairing damaged liver tissue through transplantation or cell therapy. Transplanting liver organoids, or mature hepatocytes derived from them, into the liver of patients with traumatic liver injury can accelerate liver repair by promoting hepatocyte proliferation, differentiation, and tissue regeneration.

Hemochromatosis is an abnormality in iron metabolism that results in excessive iron accumulation in the blood (138). This surplus iron is deposited in various organs, with the liver being the primary site of storage. As a result, liver lesions are a major cause of complications such as unexplained cirrhosis, bronze skin, and diabetes, with an increased risk of subsequent hepatocellular carcinoma (139). In clinical practice, diagnosis is confirmed through a positive genetic test (140). Treatment is primarily aimed at reducing blood iron levels through methods such as phlebotomy and iron chelation therapy. Patients with hemochromatosis often experience pathological changes in the liver, including damage, inflammation, and fibrosis, which can progress to liver failure (138). In this case, liver organoids offer a valuable tool for simulating and evaluating the effects of iron overload on hepatocyte function, as well as exploring potential restorative therapies through cell-based approaches.

Wilson’s disease is a genetic disorder characterized by impaired copper metabolism, leading to the accumulation of copper in the body (44). This copper overload can cause damage to the liver, nervous system, and other organs, with the liver being the most significantly affected organ in patients (44). Chronic copper accumulation results in liver damage, inflammation, fibrosis, liver failure, and even hepatocellular carcinoma (141, 142). The primary treatments for Wilson’s disease include pharmacological interventions, such as copper chelators, and liver transplantation (143). However, these therapies do not fully restore liver function and may be associated with side effects. In cases of advanced liver damage or liver failure, liver transplantation is often the only viable treatment option (144). Liver organoids hold significant potential in regenerative medicine, offering a promising approach to repair liver damage. These organoids can expand and differentiate into large numbers of functional hepatocytes *in vitro*, making them a potential alternative to liver transplantation or a “biological patch” for partially damaged liver tissue. Furthermore, the strong differentiation and proliferative capacity of organoids provide a novel strategy for promoting liver repair and regeneration in patients with Wilson’s disease.

Autoimmune hepatitis (AIH) is a chronic inflammatory liver disease caused by an abnormal immune response, characterized by immune cell attacks (especially T cells) on the liver, leading to liver damage, inflammation, and fibrosis (145). If untreated, AIH can progress to liver failure, cirrhosis, and even hepatocellular carcinoma (40). AIH is strongly associated with human leukocyte antigen (HLA) genes. The primary susceptible genotype in the European population is HLA-DRB10301, while the secondary susceptible genotype is HLA-DRB10401 (146). The pathophysiology of AIH involves an autoimmune attack on liver cells triggered by T cell activation (145). As a result, prednisone and azathioprine are commonly recommended as first-line treatments (147). In the chronic stage of AIH, the liver may develop pathological changes, including fibrosis and connective tissue hyperplasia, which can ultimately lead to liver failure (40). The regenerative potential of liver organoids offers a promising new avenue for treating AIH. By transplanting liver organoids derived from the patient or a donor, or by inducing the proliferation of liver organoids *in vitro* and directing them to the site of liver damage, liver tissue regeneration can be promoted, potentially restoring liver function. Additionally, liver stem cells or differentiated hepatocytes within the organoids can contribute to tissue repair and mitigate the progression of fibrosis.

Drugs are absorbed from the gastrointestinal tract, enter the liver through the portal vein, and are metabolized by liver enzymes (148). Unmetabolized drugs are then transported throughout the body via the bloodstream, exerting their effects on target organs (149). In recent years, the incidence of drug-induced liver injury (DILI) has gradually increased, partly due to the diminished efficacy of drugs caused by immune detection inhibitors, chemotherapy agents, polypharmacy, and adverse reactions resulting from interactions during drug metabolism (18). The Roussel Uclaf Causality Assessment Method (RUCAM) is widely employed to assess the causality of DILI, and it is crucial to discontinue the causative drug (150). Common drugs associated with DILI include acetaminophen and carbon tetrachloride, while specific drugs such as isoniazid, itraconazole, and oral contraceptives are also implicated. A small subset of DILI cases may result in chronic liver damage even after discontinuation of the causative drug (151). Autoantibodies, such as antinuclear antibodies (ANA), may become positive during DILI, complicating the diagnosis as they can overlap with AIH (152). Both conditions may present with positive ANA, elevated immunoglobulin G, and similar histopathological findings (153). The key to differentiating DILI from AIH lies in whether liver inflammation resolves after stopping the causative drug (153). In DILI, treatment primarily involves cessation of the offending drug, with supportive therapies such as ursodeoxycholic acid (UDCA) to promote bile secretion, enhance antioxidant activity, and protect the liver (154). Zhang et al. (72) demonstrated that dispersed human liver organoids (HLOs) derived from the iPSC system exhibit DILI prediction capabilities comparable to intact HLOs, with the ability to measure IC_50_ values for compound cytotoxicity. In regenerative medicine, liver replacement therapy becomes crucial when acute liver damage caused by DILI leads to liver failure. As a cell model with a high degree of self-organization, liver organoids can stimulate the proliferation and regeneration of hepatocytes through specific growth factors and signaling pathways. Transplanting liver organoids or their derivatives could potentially restore damaged liver function, offering a promising treatment option for severe drug-induced liver injury.

For patients with advanced liver cancer, liver transplantation remains the preferred treatment option (155). Current transplantation methods are categorized into orthotopic liver transplantation, heterotopic liver transplantation, and various forms of cell therapy. Cell therapy itself is further divided into hepatocyte transplantation (HTx), nonparenchymal hepatocyte transplantation, hepatic progenitor cell (HPC) transplantation, and bioartificial liver (BAL) trials (156). However, the global shortage of liver donors and the high cost of transplantation mean that many patients either cannot access a liver transplant or cannot afford the procedure, often resulting in death (155). HTx faces challenges due to issues with engraftment and host immune responses (157). Nonparenchymal cell transplantation, while used to regulate immune function and hepatocyte survival, has not yet demonstrated substantial clinical progress (89). The safety and efficacy of HPC transplantation are controversial, partly due to the role of HPCs in liver cancer development (158). Although effective in short-term liver support and detoxification, BAL devices have serious adverse reactions and do not improve long-term survival rates, limiting their application (159). Liver organoids can be transplanted into various sites, including the liver, mesentery, renal capsule, and omentum, with common methods being spleen injection, portal vein injection, and liver implantation (62, 131, 156, 160). In this context, combining liver organoids with 3D printing technology and biomaterials to create hybrid artificial livers holds promise as a potential alternative to traditional liver transplantation (161).

Liver cell transplantation necessitates long-term proliferation, sustained liver function activity, cell maturation, angiogenesis, and minimal immune rejection. Sgodda et al. (162) reported a scalable three-dimensional suspension culture system that allowed PSC-derived liver organoid cells to maintain a stable gene expression profile and metabolic characteristics for up to 3 weeks. By 2018, researchers demonstrated that human hepatocyte-derived liver organoids could proliferate for several months and exhibited successful outcomes when transplanted into mice undergoing liver resection (74). Further advancements in 2019 saw Mun et al. (163) culture liver organoids with stable liver function and long-term proliferation using PSCs. Regarding cell activity and functional stability, liver organoids composed of hepatocytes and bile duct cells generated using honeycomb micropores based on polydimethylsiloxane showed significant improvements in cell albumin secretion, liver marker expression, cytochrome P450-mediated metabolism, and bile transport function (164). Reza et al. (165) produced liver organoids that enhanced bilirubin metabolism, and portal vein transplantation in rats alleviated symptoms in a Crigler–Najjar syndrome model.

In general, iPSC-derived liver organoids are less mature than adult liver cells (166). To address this issue, liver bud (LB) cells were exposed to dexamethasone, which stimulated the BMP, FGF, HGF, and Wnt signaling pathways while inhibiting the TGFβ pathway, leading to the development of mature hepatocyte-like cells (HLCs). These cells were then used to model mature liver organoids *in vitro* and further matured into functional hepatocytes and bile ducts (167). Incorporating 3D printing technology, hiPSC-derived hepatoblasts were encapsulated in preformed aggregates of alginate beads to create human-engineered livers that closely mimic human liver function (127). This approach generates mature, functional hepatocytes and offers a permanent, unlimited source of liver cells (168). In liver organoid transplantation, the formation of blood vessels is crucial for ensuring an energy supply and metabolic circulation. Microfluidic chips have been used to simulate conventional liver transplantation within microvascular beds cultured *in vitro* l (99). Following the transplantation of liver microtissues, these microvascular beds were successfully anastomosed to establish a stable, perfusable vascular network (99). Additionally, Takebe et al. (169) developed vascularized and functional human livers from human iPSCs (iPSC-LBs) and implanted them into a mouse model of lethal liver failure. The liver buds integrated with the host blood vessels within 48 h, resulting in significant improvements in mouse survival (169). Therefore, the application of liver organoids in regenerative medicine holds tremendous promise.

### Challenges ad future of liver organoids

For liver organoids, the absence of an immune microenvironment, large blood vessels, and nerves remains a significant barrier to their broader application. In addition to these, we also need to pay attention to the standardized generation process of liver organoids, preclinical validation and regulations, interdisciplinary cooperation in approval, and industrial support. Immune rejection is a major challenge in organ transplantation, particularly in liver and kidney transplants (170). When rejection occurs, the recipient’s immune system identifies the transplanted organ as foreign and mounts an attack against it (171). To mitigate this risk, immunosuppressants are used to dampen the recipient’s immune response (172), but this approach increases susceptibility to infections and other complications (173). Thus, achieving a balance between effective immunosuppression and minimizing rejection is crucial for successful posttransplant management.

Advances in human leukocyte antigen (HLA) matching technology and transplant immunology have significantly improved the prognoses of transplant recipients (174). Although excellent HLA matching can substantially reduce rejection rates, it cannot entirely eliminate the risk (174). In organoid cultures, constructing blood vessels is a key challenge. Blood vessels are essential not only for material transport but also for maintaining tissue structure and function (175). The absence of large blood vessels can result in an inadequate nutrient supply to the organoid’s central areas, adversely impacting its development, functional expression, and long-term viability (176, 177). The absence of innervation significantly impacts liver regulation, an aspect that remains challenging within the scope of current orthotopic liver transplantation techniques. The autonomic nerves and sensory fibers within the liver’s nervous system are crucial for regulating liver function, regeneration, and disease (178). The absence or dysfunction of these nerves can significantly disrupt normal liver regulation, potentially affecting its metabolic function, regenerative capacity, and disease resistance (179). The neural activity of the liver is influenced by factors such as food intake, emotional states, and physical activity. Hepatic encephalopathy, a neurological disorder resulting from liver dysfunction, can be triggered by various conditions, including infection, gastrointestinal bleeding, electrolyte and acid–base imbalances, and drug use (180). Inflammatory responses, alterations in ammonia and lactate levels, changes in neurotransmitter concentrations, and modifications in metabolic pathways can impact nervous system function (181).

The liver communicates with the central nervous system through the autonomic nervous system, with nerve fibers playing a role in regulating liver function and response. The liver may influence the regulation of food intake and other physiological behaviors. Following liver organoid transplantation, it is imperative to simulate the electrical signals of both the autonomic and sensory nerves to maintain the body’s fluid balance. The liver nervous system is integral to liver development, regeneration, and disease processes. Strategies to enhance liver organoid function post-transplantation through the simulation of liver nervous system electrical signals—particularly regarding fluid and immune regulation—warrant further investigation.

In the context of the posttransplantation responses of liver organoids, CRISPR/Cas9 technology has been employed to create 3D biomanufactured liver structures from HLA-edited hiPSCs. This manipulation of HLA molecules results in immune-tolerant or customized liver tissues, enhancing donor-recipient compatibility and reducing the risk of acute and chronic rejection reactions (182). The rapid advancement of biomaterials and 3D printing technology is poised to revolutionize the medical field, particularly in the fabrication of artificial organs. These technologies have enabled the creation of artificial liver models with intricate vascular structures (183).

Researchers at Peking Union Medical College Hospital have successfully developed a novel artificial liver featuring a hepatic venous structure, utilizing suspension printing technology and holographic lattice acoustic tweezers (184). This development opens new avenues for alternative sources in liver transplantation (184). This model not only replicates the structural features of the liver but also demonstrates significant functional potential. Additionally, it offers a reference method for constructing an artificial liver vascular system integrated with organoids.

Integrating liver organoids into a bioartificial liver involves replacing liver cells in an artificial liver device with liver organoids and combining these with the filtering, adsorption, detoxification, and other functions of a nonbiological artificial liver (185). This hybrid approach aims to leverage the advantages of liver organoids to simulate the liver’s natural functions, thereby supporting or replacing the functions of a damaged liver. This *in vitro* device mimics the liver’s metabolic and detoxification functions by processing nutrients and metabolic waste from the gastrointestinal tract and venous blood through a semipermeable membrane, ultimately returning these substances safely to the patient’s body. It functions similarly to the *in vitro* hemodialysis equipment used for renal failure. The advantage of using liver organoids lies in their ability to replicate the cellular heterogeneity, structural functionality, and microenvironment of *in vivo* liver tissue. This maximizes the restoration of authentic liver function and helps prevent rejection and vascular embolisms associated with foreign body implantations. Another advantage is the monitorability and maintainability of function: unlike traditional transplanted organs, the activity and functionality of artificial organoid livers can be rigorously assessed before and after transplantation to ensure that they are in optimal condition prior to patient implantation. This monitoring includes evaluating the metabolic activity, protein expression, and cell signaling of liver cells to confirm proper functionality posttransplantation.

Surgery is crucial in treating liver malignancies; however, advanced-stage liver disease often necessitates the removal of a substantial portion of the liver due to systemic intolerance, severe liver damage, or a large tumor size. In such cases, the remaining liver may temporarily be insufficient to support the body’s metabolic functions postsurgery, necessitating additional supportive care to maintain vital signs. Liver organ transplantation is an effective treatment for end-stage liver disease; however, it carries risks, including immune rejection and vascular embolism (186). To mitigate these issues, artificial liver organoids and liver integrants are anticipated to replace traditional transplantation methods. These can be selected and customized based on the patient’s specific needs to minimize immune rejection.

The advancement of CRISPR/Cas9 technology offers a novel approach by enabling precise editing and customization of liver organoid cells at the molecular level, providing more targeted treatment options. Utilizing CRISPR/Cas9 technology allows for the correction of genetic defects at the cellular level or the introduction of specific functional genes. These engineered livers can more effectively adapt to the patient’s physiological environment, thereby reducing the likelihood of rejection. This technology maybe can also enhance the liver’s regenerative capacity and reduce its reliance on prolonged proliferation. Consequently, gene-edited liver cells can be expanded *ex vivo* and then transplanted into the patient, where they can continue to grow and function without eliciting an excessive immune response.

Currently, liver organoids production is typically conducted in small-scale laboratories, while clinical translation necessitates standardized, large-scale production technologies, including automated culture systems, culture medium optimization, and organoid quality control. A quality control system must be established for liver organoids to ensure that their functionality, structure, and stability meet the standards required for clinical application. To ensure the safety and efficacy of liver organoids in clinical applications, extensive preclinical validation is required. Such studies include the use of animal models, long-term safety testing, and comparative analysis of traditional models. Clinical application of liver organoids must comply with local ethical and legal requirements. This process requires enhanced collaboration with regulatory agencies, as well as the pre-design of clinical trial pathways and data collection methods to ensure the smooth approval of relevant procedures.

## Summary and outlook

The research and application of liver organoids represent a cutting-edge frontier in regenerative medicine. This technology offers novel possibilities for treating liver diseases by replicating the structure and function of the human liver. The development of liver organoids marks a significant advancement in liver transplantation technology. Traditional transplantation faces challenges such as donor shortages and rejection. Liver organoid technology offers a potential solution by reducing reliance on donor livers. Advances in biomaterials and 3D bioprinting technology are expected to enhance the structural and functional complexity of future liver organoids, bringing them closer to authentic human livers. The integration of these technologies will not only enhance the maturity and functionality of organoids but also facilitate the rapid and efficient establishment of comprehensive liver organoid models *in vitro*. This advancement has significant implications for treating end-stage liver disease, studying liver pathogenic mechanisms, and conducting drug screening. In clinical applications, liver organoid technology offers physicians a broader range of treatment options. These organoids can reconstruct the hepatobiliary system both *in vivo* and *in vitro*, demonstrating significant potential for liver function replacement and tissue regeneration. Particularly in *in vivo* transplantation, liver organoids have shown the potential for safe and effective implantation, with demonstrated therapeutic effects. Moreover, combining liver organoids with other tissue-engineering materials may further enhance their therapeutic efficacy.

In summary, liver organoid technology has broad application prospects in regenerative medicine. Although liver organoid transplantation has shown promising results at the laboratory stage, its efficacy has been demonstrated in mice, pigs, and other animal models. This technology holds significant potential. However, the clinical application of liver organoid transplantation has not yet been reported, and crucial evidence-based clinical research remains insufficient. As related technologies continue to advance, more personalized and precise treatment plans for liver disease are anticipated, offering renewed hope to patients. Progress in this field could not only enhance the quality of life for patients with liver disease but may also fundamentally transform our understanding and treatment approaches for liver disease.
